# Body condition reveals hidden correlations between co-infection and behavior in sunfish

**DOI:** 10.1093/beheco/araf055

**Published:** 2025-05-24

**Authors:** Victoria Thelamon, Frédérique Dubois, Maryane Gradito, Sandra A Binning

**Affiliations:** Département de sciences biologiques, Université de Montréal, 1375 Av. Thérèse-Lavoie-Roux, Montréal, Québec, H2V 0B3, Canada; Département de sciences biologiques, Université de Montréal, 1375 Av. Thérèse-Lavoie-Roux, Montréal, Québec, H2V 0B3, Canada; Département de sciences biologiques, Université de Montréal, 1375 Av. Thérèse-Lavoie-Roux, Montréal, Québec, H2V 0B3, Canada; Département de sciences biologiques, Université de Montréal, 1375 Av. Thérèse-Lavoie-Roux, Montréal, Québec, H2V 0B3, Canada

**Keywords:** behavioral trait, cognition, freshwater fish, fulton condition index, host/parasite interactions

## Abstract

The role of parasites in maintaining consistent inter-individual differences in behavior (ie personality) is the subject of increasing study and debate. While behavioral differences may expose individuals differently to parasites, parasite infection can itself change host behavior, sometimes favoring the parasite’s own transmission. Furthermore, parasites can alter the functioning of energetically costly organs like the brain, thus affecting cognitive performance. However, relationships among infection, cognition, and behavior can be complex and difficult to interpret, especially in wild populations where individual health status is unknown. The inclusion of body condition as a fitness proxy may help reveal relationships between parasites and host traits that are otherwise masked. We examined relationships among host body condition, personality (ie exploration, boldness), cognition (ie aversive learning) and parasite density in wild-caught pumpkinseed sunfish (*Lepomis gibbosus*) naturally infected with endoparasites. Exploration in an open field test was repeatable in sunfish. Boldness, assessed using a shelter test, was not repeatable, but was correlated with exploration. Host exploration decreased with both increasing parasite density and decreasing body condition. Only individuals in relatively lower body condition displayed a negative relationship between parasite density and exploration, suggesting a pathologic effect of the parasites on the sunfish. Aversive learning was not influenced by co-infection. Our results show that body condition is important to consider when studying wild populations as some patterns observed between parasite density and host behavior were only revealed when body condition was taken into consideration.

## Teaser

We measured behavioral traits and aversive learning in naturally co-infected pumpkinseed sunfish. We found that body condition, a measure of health, revealed links between behavior and parasite infection. Only relatively less healthy individuals with lower body condition displayed a negative relationship between parasite density and exploration. Aversive learning was not influenced by co-infection. Our study highlights the importance of including covariates in revealing hidden correlations between behavior and infection in wild animals.

## Introduction

Experimental studies in controlled laboratory conditions are critical for establishing causal relationships between behavior or cognitive performance (ie all mechanisms enabling individuals to acquire, process, store and use information) and environmental stressors. However, recreating natural conditions in laboratory settings is often impossible, hampering ecological inferences. In particular, biological factors, such infection with parasites (eg organisms that live and use resources on or in a host; Goater et al. 2014), are difficult to reproduce in the lab. For instance, natural microbial communities (Kohl and Dearing 2014) or infection with multiple parasites at the same time (ie co-infection) (Venter et al. 2022) can be difficult to reproduce experimentally because infective parasite stages may not be possible to manipulate in the lab, the order of host colonization by parasites may be important for host impacts, and the intensity of experimental infections may not reflect those present in nature (Sharp 1990; Walaza et al. 2020). These difficulties are particularly pronounced for parasites with complex life cycles (ie requiring multiple hosts for development) (Hoffman and Putz 1965). This is problematic given that wild animals are commonly infected with multiple parasites simultaneously, thus an understanding of parasite impacts on host behavior and cognition following experimental infection in the lab may not accurately represent the complexities of co-infection, which is more akin to reality (Mousquer et al. 2021). Leveraging natural infection gradients in wild-caught animals could help provide insights into the links between behavior, cognitive performance and parasitism in more natural settings. For instance, plumage color and parasite load in barn swallows (*Hirundo rustica erythrogaster*) were linked with multiple parasite associations, and different patterns emerged (positive, negative and neutral) between sexual coloration and co-infection within one population (Hund et al. 2021), showing the importance of considering the natural parasite fauna of wild animals. Studying wild populations may also provide more realistic insights into the extent of individual variation in cognitive and behavioral traits across an infection gradient.

Parasites are increasingly recognized for their important role in maintaining consistent inter-individual differences in host behavior (ie personality) and cognition in nature (Shettleworth and Hampton 1998; Kortet et al. 2010; Poulin 2013; Barber et al. 2017; Dubois and Binning 2022). Parasites may alter host behavior through various processes such as sickness behavior (eg increased lethargy during acute phase of infection; Lopes et al. 2021), parasite manipulation (eg increased boldness favoring parasite transmission to final hosts; Berdoy et al. 2000; Poulin 2010; Hafer and Milinski 2016) or through the host compensatory response (eg increased foraging behavior to compensate for energy loss caused by the parasite; Lefèvre et al. 2008). In contrast, a growing body of studies suggest that behavior can also influence how wild individuals interact with their environment and thus, make them more or less susceptible to infection (Koprivnikar et al. 2012; Gradito et al. 2024; Payne et al. 2024). A pioneering study by Wilson et al. (1993) suggested that pumpkinseed sunfish (*Lepomis gibbosus*) vary in their individual behavior, which leads them to interact differently with their environment in ways that influence the acquisition of trematodes. Furthermore, as parasites impose costs on their hosts, infection should compromise the functioning of energetically costly organs such as the brain. Indeed, studies on mice (Kavaliers et al. 1995; Hodkova et al. 2007), honeybees (Iqbal and Mueller 2007) and fishes (Binning et al. 2018) have found that cognitive performances are negatively affected by experimental and/or natural parasite infection. Unlike in the animal personality research, no study has yet shown manipulation of the host’s cognitive abilities to favor parasite transmission, such as altering the host ability to learn to avoid certain habitats or to suppress certain behaviors (Ducatez et al. 2020). That said, inter-individual variation in cognitive abilities can also potentially impact parasite load, as hosts can learn to avoid or respond to parasites differently. Despite growing interest in this field, the majority of studies on cognition and parasite infection focus on laboratory populations of rodents and insects with few studies in wild populations across other taxonomic groups (Ducatez et al. 2020; Salena et al. 2021).

Although studying wild-caught animals harboring natural infection gradients is ecologically realistic, it can be difficult to detect correlations among variables of interest. Indeed, some correlations can be hidden when covariates are not included, or positive correlations may appear where we would expect a considerable cost at the individual level (Van Noordwijk and de Jong 1986; Reznick et al. 2000). Although parasitism is expected to be negatively correlated with host fitness traits and health metrics at the population level, parasite load and body condition (ie physical state of an individual; Bolger and Connolly 1989) can be positively correlated at the individual level (Sánchez et al. 2018). Thus, including a covariate, such as body condition, when exploring correlations between parasitism and behavior in wild-caught animals can reveal critical trade-offs in hosts as a result of differences in their body condition (Alzaga et al. 2008; Klemme et al. 2020). For instance, dispersal in juvenile roe deer (*Capreolus capreolus*) decreases with both increasing parasite abundance and decreasing body mass (Debeffe et al. 2014). Studies in birds (Shaw 2017) and primates (Huebner et al. 2018) have also linked measures of cognition with a fitness proxy like body condition. Therefore, including a measure of body condition as an index of host health in wild populations may reveal relationships among behavior, cognition and parasite load that would otherwise remain hidden if these same questions were investigated on laboratory populations, where subjects are typically well fed, protected from predation, in stable environments and in overall good condition.

**Fig. 1.**
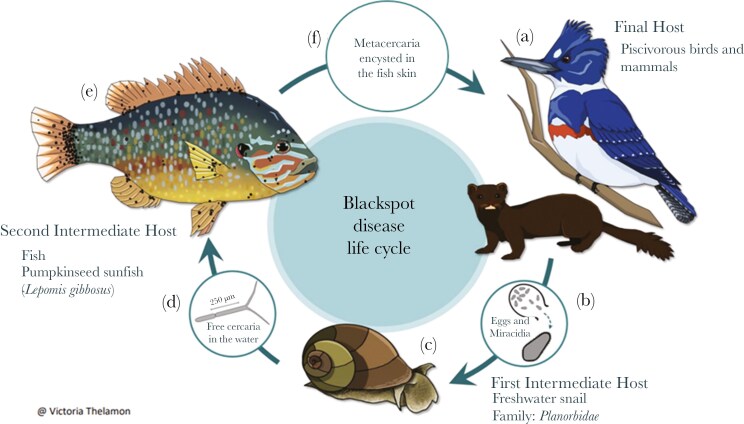
Simplified life cycle with first and second intermediate hosts and the final host of parasites causing blackspot disease in fish in North America (trematodes of the genus *Uvulifer sp*., *Crassiphalia sp*. or *Apophallus sp*.). (A) After sexual reproduction in the gut of the final host, eggs are released into the water in feces. (B) Eggs hatch and release miracidia that infect a freshwater snail. (C) Miracidia penetrate the soft tissues of a snail and multiply asexually. (D) Released cercariae swim freely and contact a host fish (Cypriniformes, Esociformes, Perciformes and Salmoniformes). (E) Encystment of the cercaria (which has become metacercaria) creates a visible black spot on the skin of the host fish. (F) Final host infection occurs through ingestion of the second intermediate host. Yellow grub (*Clinostomum marginatum*) life cycle consists of the same steps as the blackspot species. Image created by V. Thelamon.

Here, we explored relationships among parasite infection, host behavior (ie exploration, boldness), cognitive performance (ie aversive learning) and body condition in wild-caught pumpkinseed sunfish (*Lepomis gibbosus*) naturally co-infected with endoparasites with complex life cycles. Pumpkinseeds are often heavily infected with multiple endoparasites including trematodes and cestodes in the wild (Chapman et al. 2015) and previous studies have measured boldness, exploration, and cognition in wild-caught individuals (Wilson et al. 1993; Gradito et al. 2024). In Quebec’s Laurentian region, pumpkinseeds are commonly infected by two species of trematodes (eg *Uvulifer ambloplitis* and *Apophallus sp.*) causing visible blackspots on the fish skin (ie blackspot disease) (Fig. 1) and one cestode, the bass tapeworm *(Proteocephalus ambloplitis)* causing liver disease Archambault et al. (2025). We predict that a higher parasite density (ie more parasites per gram of body mass) will affect host’s behaviors (ie more exploratory and bolder individuals either to compensate for the energetic costs of parasite infection and/or parasite manipulation). Furthermore, fish with higher parasite density should be more negatively affected than less parasitized fish in an aversive learning task. Finally, the inclusion of body condition as a proxy for individual health status could provide insights into correlations between parasitism, behavior and cognition. If parasites influence the behavior and/or cognitive performances of their hosts to ensure their transmission to definitive hosts, we expect that the impact of parasites on the exploration, boldness and aversive learning ability of hosts will be stronger for fish in lower body condition (ie significant interaction between body condition and parasite densities). Since pumpkinseed sunfish are co-infected with multiple parasites, we also expect potential conflict between parasites in the host, which may lead them to alter different aspects of host behavior: we expect to find interactions between both parasite densities for boldness, exploration and cognitive performance.

## Methods

### Ethical note

Fish were collected and cared for with approval from the Université de Montréal’s animal care committee (Comité de déontologie de l’expérimentation sur les animaux; permit number 19-034) and the Ministère des Forêts, de la Faune et des Parcs (collection permit number 2019-05-17-1580-15-S-P).

### Animal collection and husbandry

This study was conducted at the Station de Biologie des Laurentides (SBL, Québec; 45.98898°N, -74.00013°W) de l’Université de Montréal between June and September 2019. A total of 90 pumpkinseed sunfish (*Lepomis gibbosus*) were collected with a seine net in Lake Cromwell (45.59231°N, -73.59565°W). Only subadult sunfish were kept (mean mass: 10.71 ± 3.34 g, mean standard length: 69.62 ± 7.03 mm). Within an hour of capture, subjects were transported in aerated plastic buckets to the SBL laboratory facilities (apx. 350 m distance from collection site) and were treated in a hydrogen peroxide bath (2.5 ml of 3% H_2_O_2_ per liter of freshwater) for 30 min to prevent fungal and bacterial outbreaks that sometimes occur in this species in captivity. Unfortunately, twenty-six fish developed skin infections despite this treatment and died during the habituation period. Thus, behavioral and cognitive tests were performed on 65 individuals. No noticeable surface infections or ectoparasites were visible on the tested fish. Fish were then transferred to a 600 L flow-through holding tank (215 × 60 cm and 60 cm high) partitioned in three equal sections, with PVC tubes provided as shelters. Each section contained a group of 15 to 20 subjects. Water pumped directly from nearby Lake Croche (45.99003°N, -74.00567°W) was particle-filtered, oxygenated and UV-sterilized prior to entering the holding tank. Water temperature was maintained at 21°C. Light followed a 16:8 light to dark cycle. Temperature and oxygen concentration in the tank were monitored daily and adjusted as needed. The tank was siphoned once a day to remove uneaten food and waste.

Twenty-four hours after capture, fish were measured (standard length in millimeters) and weighed (in grams) to measure body condition. They were also uniquely marked with visible elastomer implant tags (VIE; Northwest Marine Technology) on the side of the dorsal fin with a three-color code (combination of blue, red, green, pink and/or orange). Fish were left to acclimate in their holding tank for at least three days before behavior and cognition tests (see below). Fish were fed to satiation twice a day with bloodworms and commercial cichlid pellets (Nutrafin Bug Bites). Fish were fasted for at least 12 h prior to any experiment to reduce food rejection due to stress. Fish were euthanized following behavioral and cognitive tests with an overdose of eugenol and kept in a freezer at -18 °C until dissection. During dissection, fish were remeasured, inspected for parasites and sexed. The number of blackspots (eg trematodes that encyst in the fish skin and create visible blackspots; *Apophallus* sp and *Uvulifer sp.*) on the body, fins and gills were counted on the left side of each fish only to avoid double-counting parasites. Blackspot infection is equally likely to appear on the right vs. left side of an individual with no significant difference between the number found on either side of a fish (De Bonville et al. 2024). Each fish was also examined for other endoparasites (eg *Clinostomum marginatum*, *Posthodiplostomum sp and Proteocephalus ambloplitis*) encysted in muscle tissues and/or present in the body cavity, internal organs and digestive tract.

### Behavioral experiments

All behavioral trials were conducted between 9:30 and 12:00. Through all handling procedures, fish were manipulated in an individual plastic bag filled with water, to minimize air exposure and stress. For behavioral tasks, plastic bags were covered in black adhesive to reduce external disturbances during the transport from the holding tank to the experimental apparatus. Experimental tanks were filled with the same water as the holding tanks, which were also maintained at 21°C. Water in experimental arenas was changed between each fish.

### Personality tests

In order to assess personality traits, all individuals performed two tests: an open field test and a shelter test, in tanks containing 25 liters of water (tank: 60 × 41 cm and 34 cm high, 10 cm of water). Tank sides were covered on the exterior with white adhesive to minimize external disturbances. All tests were filmed from above with a digital camera (GoPro Hero 4 Black).

A modified open field/shelter emergence test was used to measure the percentage of surface explored by an individual (ie exploration) (Réale et al. 2007; Gibelli et al. 2019) as well as the latency of an individual to emerge from a shelter after being chased (ie boldness) (Brown et al. 2005). All fish were placed on the right side of the tank at the start of the trial and left undisturbed for 15 min. The first 10 min were used to estimate exploration (ie percentage of surface area covered in 10 min). No data was recorded during the subsequent 5 min. Together, this 15-minute period was also used as habituation to the tank for the shelter test. The shelter used was a 20 cm long white PVC tube placed on the left side of the experimental tank before the fish was added. After the 15 min of habituation, the fish was chased by a hand net until it entered the shelter (fish who entered the shelter; trial 1 = 26; trial 2 = 29) or for a maximum time of 10 s (fish who did not enter the shelter; trial 1 = 38; trial 2 = 35). Trials lasted a maximum of 300 s. All fish performed both tests in the same order, open field and then shelter test. Both tests were repeated twice, with at least a 4-to-6-day delay between each test.

The percentage of tank surface explored by the fish in 10 min during the open field test was extracted using MATLAB. The latency to emerge from the shelter was extracted by video playback using VLC media player. Videos were renamed by a colleague (not an author) and analyzed in a randomized order by VT who was blind to the individual and trial number to reduce observer bias. Boldness was assessed as the latency to emerge completely from the shelter (ie time for the whole body, including the tail to leave the shelter). For the individuals that did not use the shelter, we instead used the time to swim out of the 10 × 20 cm zone it occupied (similar size to the shelter) as our measure of latency. The latency period was recorded from the moment the fish stopped moving (ie freeze) until it swam out of the 10 × 20 cm zone.

### Learning task: inhibitory avoidance learning

**Fig. 2.**
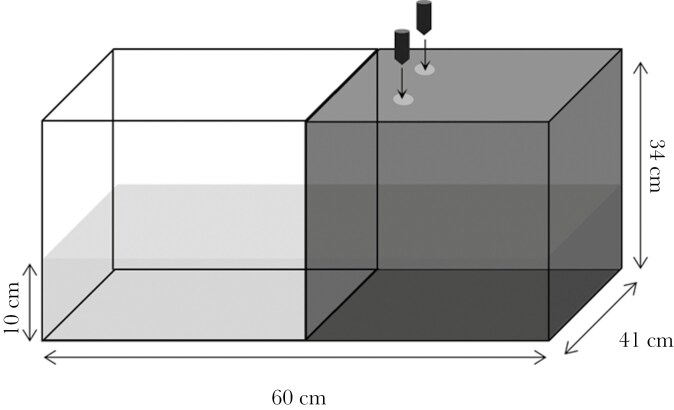
Schematic representation of the inhibitory avoidance set up, with a white and black side. Compartments were distinguished with adhesive in black or white, covering the walls and the bottom of the tank. The black side was covered with a black tainted see-through Plexiglas, with two holes that allowed two electromagnets to drop in the tank. Electromagnet release was controlled manually by the experimenter.

We measured the fish’s ability to associate a preferred environment with a threat and to subsequently avoid it (Blank et al. 2009). Trials were conducted 8 d following the behavioral tests in a similar tank as described above. However, the tank was divided widthwise into two zones: a white side (left) and a black side (right) (Fig. 2). Most fishes including sunfish will naturally swim towards the darkest part of the environment, because it is considered less of a threat (Blank et al. 2009). Thus, the black side was considered the preferred side of the tank. The black side was covered with a black tinted Plexiglas cover, with two holes. The holes allowed two electromagnets controlled by the observer to drop in the tank to scare the fish (Fig. 2). Two magnets were dropped simultaneously, to ensure that fish were startled upon release of the weight regardless of where they entered the zone. All tests were filmed from above with a digital camera (GoPro Hero 4 Black). Fish movement was visualized remotely through live feedback on a smartphone connected to the digital camera via the GoPro app (Version 6.1) to determine the moment of electromagnet release.

Trials proceeded as follows: subjects were acclimated for 3 min in a 500 ml glass container placed on the white side of the tank. The fish was then gently released into the aquarium and time recording started. The magnets were dropped as soon as the subject had completely crossed (whole body including tail) to the black side. The trial was repeated three times with a 24-hour delay. The first trial was used as the learning phase. The two subsequent trials were the test phases. After the first trial (ie learning phase), the fish should learn that the black side represents a danger. A maximum time of 180 s was given to allow the fish to enter the black side before the trial was stopped. Data was extracted by video playback using VLC media player. Latency (in seconds) to enter the black side on all trials was measured. We used the absolute difference between trial 2 and 1 (following the learning phase) in the time to enter in the black side as our measure of cognitive performance. Only one fish did not participate in the first trial. For this fish, we used the latency on the third trial for the analysis.

In the learning task, it is possible that more anxious fish cross to the black side faster than less anxious fish, thus we added this variable in our model for cognitive performance (Maximino et al. 2010; Gibelli et al. (2019)). Anxiety was measured as the time spent in a preferred environment (ie the black side) compared to an unpreferred environment (ie the white side). The test was conducted before the inhibitory avoidance learning experiment in a similar tank used for the inhibitory avoidance learning experiment. Fish were placed on the white side of the tank and left undisturbed for 15 min. Data was extracted by video playback on VLC. Time spent in the black side was measured for the first 10 min, the timer was paused when half the body of the subject was in the white side. Percentage of total time spent in the black side was used in the analysis.

### Statistical analysis

Statistical analyses were performed with *R* 4.0.2. The level of statistical significance was set at α = 0.05 for all analyses. Adjusted fish mass was calculated as the total fish mass minus parasite mass (Lagrue and Poulin 2015). Parasite mass was determined by calculating the mean mass of 20 parasites of each species detected and then divided by 20 to obtain an estimated mass for 1 parasite (yellow grub (*Clinostomum marginatum)*: 0.007 g; bass tapeworm (*Proteocephalus ambloplites)*, adult: 0.003 g, larval form: 0.0008 g). The mass of trematodes causing blackspots (ie *Apophallus sp*. or *Uvulifer sp*.) was too small to be subtracted from the fish total mass (10^-7^ g). Because of a freezer malfunction, 21 fish were accidentally defrosted and decomposed before internal parasites could be counted. We used n = 65 to look at repeatability and correlations among behavioral and learning traits as all fish were tested but our final sample was reduced to n = 44 for models looking at the effect of parasite densities on exploration, boldness and cognition. Most fish were immature and could not be accurately sexed from gonad inspection. Thus, sex was not included as a variable in our analyses.

Fish body condition was calculated as the Fulton index (K) using the adjusted fish mass (mass/standard length^3^ in cm) (Jakob et al. 1996). Mean (± SEM) body condition was 3.09 ± 0.36. The number of blackspot-causing trematodes was not correlated with the number of other parasites found in the fish’s body cavity, muscle tissues or digestive tract (n = 44, df = 42, r = 0.11, p = 0.49). Thus, parasite density (n/g) was calculated as the number of parasites divided by the adjusted fish mass, for blackspot trematodes and internal parasites (including bass tapeworm and yellow grub) separately. Pearson’s correlations were calculated for pairwise combinations of body condition, internal parasite density and blackspot density. As the correlations between body condition and blackspot density (n = 44, df = 42, r = 0.22, p = 0.16), body condition and internal parasite density (n = 44, df = 42, r = 0.08, p = 0.61) and both parasite densities (n = 44, df = 42, r = 0.13, p = 0.39) were not significant, we included these variables in all models. Further, anxiety was not correlated with blackspot density (n = 44, r = 0.07, p = 0.653) nor internal parasite density (n = 44, r = -0.28, p = 0.07) using Spearman correlations. Anxiety was not correlated with mean exploration (n = 65, r = -0.2, p = 0.11), mean boldness (n = 65, r = 0.12, p = 0.36) or mean cognitive performance (n = 65, r = -0.15, p = 0.22), using Spearman correlations.

We tested for repeatability between trials in the measures of exploration and boldness with the *rptR R* package using 1000 bootstraps (Stoffel et al. 2017) to validate the behavioral tests with the pumpkinseed sunfish. Residuals were visually inspected for normality. We fitted the models with a Gaussian distribution for exploration (arcsin-transformed) and binary distribution for boldness. Trial, body condition, blackspot density and internal parasite density were included as fixed factors and fish identity as a random factor for each trait. To measure the correlation between all behavioral and cognitive traits, we build a GLMM using a quasipoisson family to account for the non-normality and overdispersion of the data. We measured the relationship between exploration, boldness and cognitive performance (3 GLMM in total) with ID as a random factor to account for the repeated measures. All variables were z-standardized (ie *z*-scored) prior to analysis. To quantify the effect of parasite density on exploration (response variable), we used linear mixed models with the *lme4* R package (Bates et al. 2015). Fish body condition, blackspot density, internal parasite density and trial number were included as fixed effects with fish identity included as a random factor. Percentage of surface explored was Arcsin transformed to meet model assumptions following a visual inspection of the model residuals. Body condition and both parasite densities were *z*-standardized. We added an interaction between body condition and blackspot density and one between body condition and internal parasite density to understand how a proxy for fish health interacts with infection. We also included an interaction between blackspot density and internal parasite density to explore potential conflict between parasite types on the behavior and cognition of host fish.

Data from the shelter test was highly right skewed as 40 subjects out of 65 did not emerge from their shelter during the 5-minute trial (including 11 subjects that did not emerge in both trials). To quantify the effect of parasite density on latency to emerge from a shelter, we used a generalized linear mixed model (GLMM) with a binomial error distribution (1: emerged from shelter; 0: did not emerge from shelter). Fish body condition, blackspot density, internal parasite density and trial number were included as fixed effects with fish identity included as a random factor. Body condition and both parasite densities were *z*-standardized. The same two-way interactions as above between internal parasite density, blackspot parasite density and body condition were added to this model.

To quantify the effect of parasite density on fish aversive learning, we used a generalized linear model (GLM) with a quasipoisson error distribution from the *lme4 R* package. Body condition, blackspot density, internal parasite density and anxiety were included as fixed effects. A reciprocal square root transformation (1/√x) was used on the latency difference between trial 1 and 2 to enter the black compartment to meet model assumptions. All covariates were z-standardized. The same interactions as described above were added to this model.

## Results

### Blackspots and internal parasites

Besides blackspot-causing trematodes, the two most abundant species of parasite found in our fish samples were the yellow grub (Trematoda: *Clinostomum marginatum* (Rudoplhi, 1819); prevalence: 14%, min-max: 0 to 4, median: 0), which were encysted in muscle tissues, and the bass tapeworm (Cestoda: *Proteocephalus ambloplites* (Leidy, 1887), adult and larval form; prevalence: 100%, min-max: 1 to 227, median: 22) found in the liver, abdominal cavity and digestive tract. One fish had an unidentified nematode species (roundworm) in its digestive tract. The most abundant species of trematode causing blackspots was *Apophallus* sp. (Binning, Lanthier, unpublished data), with *Uvulifer sp*, also present. The number of blackspots on the fish’s left side ranged from 0 to 270 (Prevalence: 97.7%, min-max: 0 to 270, median: 75).

### Personality tests

The percentage of the tank explored by a fish (ie exploration) was repeatable between trials (n = 65, R = 0.28, 95% CI = [0.04, 0.48], p = 0.02). However, emergence from the shelter (ie boldness) was not repeatable between trials (n = 65, R = 0.03, 95% CI = [0, 0.23], p = 0.372 for binomial data). We found a strong correlation between exploration and boldness (n = 65, cor = -0.80, p = 0.01): fish that explored more also emerged faster from the shelter. Although not significant, we found moderate correlations between exploration and cognitive performance (n = 65, cor = -0.46, p = 0.17): fish that explored less tended to perform better in the cognitive task. We also found a correlation between boldness and cognitive performance (n = 65, cor = -0.50, p = 0.2): fish that emerged faster from the shelter tended to perform better in the cognitive task.

### Open field test

**Fig. 3.**
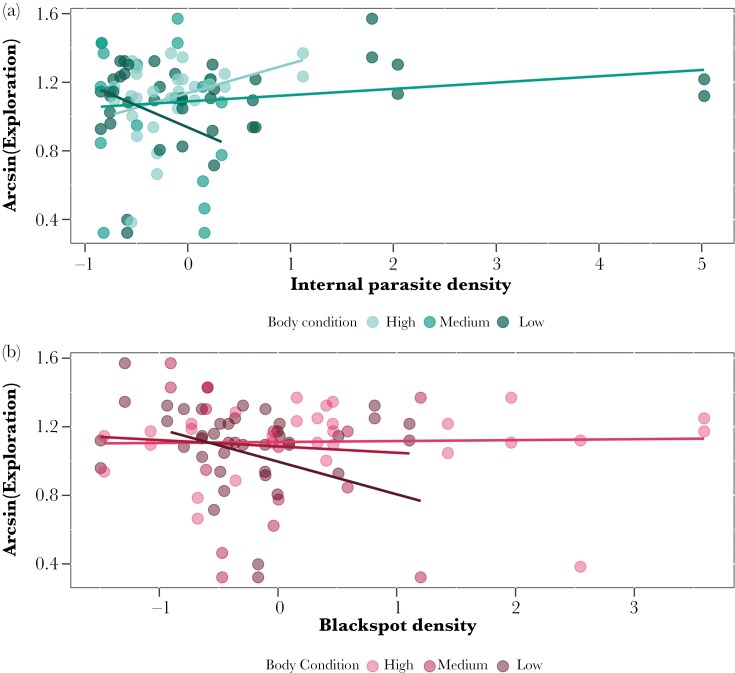
Interaction between fish body condition, exploration and internal parasite density (A) or blackspot density (B). Internal parasite density and blackspot density were z-standardized. Fish were separated in 3 groups according to their body condition level (high: n = 30, max-min = 3.38 to 3.73; medium: n = 42, max-min = 3.00 to 3.33; low: n = 16, max-min = 2.57 to 2.96) for visualization purposes using the silhouette width index (SSI) to determine the number of clusters. For (A): the darker green line represents low body condition, the light green line high body condition and medium body condition is represented by the medium green line. For (B): the dark red line represents low body condition, the bright red represents high body condition and the medium red represents medium body condition. However, the linear model included internal parasite, blackspot density and body condition as continuous variables. Each individual (n = 44) is represented twice with dots (for each exploration trial).

There was a significant interaction between fish body condition and internal parasite density for exploration (Table 1): fish in relatively better condition explored more when internal parasite density increased whereas fish in lower body condition explored less as parasite density increased (Fig. 3A). A significant interaction between body condition and blackspot density was also found (Table 1; Fig. 3B). The interaction between blackspot density and internal parasite density was not significant (Table 1). There was a negative relationship between exploration and blackspot density: fish with higher blackspot density decreased their exploration. Body condition and exploration were positively related: fish in better body condition increase their exploration (Table 1).

**Table 1.** Linear mixed model between percentage of tank surface explored (ie exploration) and body condition, blackspot density, internal parasite density and trial number as fixed effects. Fish identity was included as a random effect. All explanatory variables were z-standardized. n = 44. Estimates in bold are statistically significant. Intraclass Correlation Coefficient (ICC), marginal R squared (R^2^m) and conditional R squared (R^2^c) are included in the table.

	Estimates	T value	P value	R^2^m	R^2^c	ICC
**Exploration (arcsin)**				0.08	0.40	0.38
Body condition	0.12	2.27	**0.03**			
Blackspot density	−0.13	−2.28	**0.03**			
Internal parasite density	−0.01	−0.16	0.88			
Trial	−0.06	−1.35	0.18			
Body condition*blackspot density	0.11	2.06	**0.05**			
Body condition*internal parasite density	0.21	2.0	**0.05**			
Blackspot density*internal parasite density	−0.06	−1.4	0.17			

### Shelter test

We found a significant negative relationship between emergence from a shelter and trial number (Table 2): fish were less likely to emerge following a chase in the second trial. There was a marginally significant negative relationship between emergence and blackspot density (Table 2): fish with more blackspots were somewhat less likely to emerge from the shelter. There was a marginally significant interaction between body condition and internal parasite density on emergence (Table 2). No relationship was found between emergence and body condition nor between emergence and internal parasite density (Table 2). The interaction between body condition and blackspot density, and the interaction between blackspot density and internal parasite density were not significant (Table 2).

**Table 2.** Generalized linear mixed model with emergence from the shelter as the response variable (ie boldness) and body condition, blackspot density, internal parasite density and trial number as fixed effects. Fish identity was included as a random effect. All explanatory variables were z-standardized. n = 44. Estimates in bold are statistically significant. Intraclass Correlation Coefficient (ICC), marginal R squared (R^2^m) and conditional R squared (R^2^c) are included in the table.

	Estimates	z value	P value	R^2^m	R^2^c	ICC
**Emergence from shelter (binomial)**				0.29	0.33	0.06
Body condition	0.61	1.42	0.16			
Blackspot density	−1.16	−1.86	0.06			
Internal parasite density	0.37	0.97	0.33			
Trial	−1.02	−2.00	**0.04**			
Body condition*blackspot density	1.02	1.79	0.23			
Body condition*internal parasite density	−1.12	−1.21	0.07			
Blackspot density*internal parasite density	0.30	0.85	0.40			

### Inhibitory avoidance learning

We found no relationship between the latency difference between trial 2 and 1 (ie cognitive performance) and blackspot density, nor between cognitive performance and internal parasite density (Table 3). There was no significant relationship between cognitive performance and body condition or trial (Table 3). We found no significant relationship between cognitive performance and anxiety (Table 3). No interactions included in the model were significant (Table 3).

**Table 3.** Generalized linear model with the difference between trial 2 and 1 in time to enter the black side of the tank (ie cognitive performance) as the response variable and body condition, blackspot density, internal parasite density and anxiety (ie percentage of time spent in the black side) as fixed effects. The difference between trial 2 and 1 in time to enter the black side was reciprocally squared-transformed. All explanatory variables were z-standardized. n = 44. Estimates in bold are statistically significant.

	Estimates	T value	P value	R^2^
**1/sqrt(Inhibitory avoidance learning)**				0.12
Body condition	0.39	0.59	0.56	
Blackspot density	0.36	0.09	0.93	
Internal parasite density	-0.04	−0.01	0.99	
Anxiety	0.26	1.4	0.17	
Body condition*blackspot density	0.04	0.05	0.96	
Body condition*internal parasite density	-0.04	−0.03	0.98	
Blackspot density*internal parasite density	-0.05	−0.22	0.83	

## DISCUSSION

Pumpkinseed sunfish collected and used in this study were highly parasitized by two main species: *Apophallus sp*., a trematode causing blackspot disease, and *Proteocephalus ambloplites* or bass tapeworm, a cestode found in the abdominal cavity and digestive tract of fish (referred to here as internal parasites). No correlation was found between blackspot density and internal parasite density, suggesting that these infections are likely acquired independently from one another.

### Personality and parasitism

Exploration was moderately repeatable (R = 0.28), and we found that sunfish explored less when they had more parasites. This included infection by blackspot-causing trematodes, as well as internal parasites for hosts in relatively lower body condition. We expected a positive relationship between parasite density and exploration behavior as both cestodes and trematodes have complex life cycles and would benefit from host behaviors that increase the likelihood of predation from definitive hosts. However, endoparasites can also increase host energetic costs through altered metabolic rates (McElroy and de Buron 2014; Binning et al. 2017). As such, host fish may reduce their exploratory behaviors, especially when they are in low body condition, because this behavior is costly to sustain. Few studies have tested the effects of blackspot trematodes on host fish physiology, making it difficult to infer the energetic costs of these parasites to hosts. Once formed, blackspot cysts are metabolically inactive (Lemly and Esch 1984). Thus, fish hosts likely only experience the cost of mounting an immune response during the encystment of the cercariae (Hoffman and Putz 1965). Recent studies on infected sunfish from Laurentian lakes have been mixed with some studies finding no relationship between blackspot load and whole-organism or cellular metabolism (Guitard et al. 2022; Mélançon et al. 2023), with others documenting positive or negative relationships between trematodes and host metabolic rates (Thambithurai et al. 2022; Levet et al. 2024) and thermal tolerance (De Bonville et al. 2024). Indeed, previous studies on blackspot disease found reduced overwinter survival in juvenile pumpkinseed sunfish with more than 50 cysts, suggesting that these parasites do impose an energetic cost on their hosts at certain levels of infection (Lemly and Esch 1984). It is possible that the relationship found in this study is the result of sickness behavior. If the immune system is constantly activated by infection, then decreases in exploration could be a byproduct of host immune activation (Hart 1990).

Bass tapeworms are acquired by the fish host when they ingest infected copepods (first intermediate host) (Meyer 1962; Hoffman 1999). Once in the fish’s body cavity, they directly feed on the liver, gonads and digestive system. Organ function in highly parasitized fish can be deteriorated to the point of hepatic necrosis and sterility (Meyer 1962; McDaniel and Bailey 1974; Gillilland and Muzzall 2004; Archambault et al. (2025)); hosts may respond to this pathologic effect of infection by exploring less. However, little is known about the impact of this parasite on fish host physiology or behavior. This is surprising given that the bass tapeworm is widespread across North America and is a well-known parasite in both recreational and commercial fisheries (Hoffman 1999). In our study, infection prevalence was 100% with a median intensity of 22 tapeworms per fish. Exploration was reduced with increasing internal parasite density for individuals in low body condition, suggesting a pathological effect of this parasite on the sunfish. It has been shown that pumpkinseed sunfish infected with bass tapeworms have reduced body condition (Gradito et al. 2024) and reduced responsiveness to simulated avian predators with high tapeworm density (Guitard et al. 2022), which is coherent with our results. Body condition could be reduced by internal parasite infection, due to liver damage, which could also reduce exploration to divert energy to fight the infection. The fact that such a common and widespread parasite, with seemingly important effects on host physiology and behavior, has been so understudied underscores just how little we understand about the role of parasites in natural host populations.

Contrary to our expectations, fish with more blackspots tended to be less likely to exit the shelter than less parasitized fish. However, we cannot draw many conclusions from these results, as latency to emerge from the shelter was not repeatable across trials and as such, does not accurately represent a personality trait like boldness, even though it was significantly correlated with exploration (cor = -0.80). Further, the significant relationship between emergence and trial could indicate habituation to the manipulation/shelter test. Therefore, a more accurate measure of boldness that is repeatable across time and contexts is needed to further explore this behavioral trait in the context of parasitism in this system.

### Personality and body condition

The significant interactions between body condition and parasite density on host behavior found in this study illustrate the importance of including a measure of overall animal health when studying performance in wild populations. Individuals originating from the same population may vary in the extent to which they are impacted by parasite infection because of body condition differences. This idea is illustrated in our study by the lack of an overall correlation between body condition and parasite density, even though cestode parasite infection leads to a clear pathology in hosts (ie hepatic necrosis) (Archambault et al. 2025) . Rather than parasites being generally related to decreased host body condition, some of the interesting patterns between infection and the behavior tested were only revealed when this variable was considered in the models (Sánchez et al. 2018). The importance of body condition in revealing patterns between parasite density and exploration suggests that parasites could impact their hosts through effects on physiology: individuals in relatively lower body condition displayed a negative relationship between parasite load and exploration, which would be predicted if parasite infection causes changes in host behavior. Healthier individuals appear not to show a strong relationship between parasite density and exploration, making it less likely that personality traits themselves cause individuals to acquire more infections (Poulin 2013). In contrast, a recent study using cages in a lake to naturally infect sunfish showed that even in restricted areas like a cage, sunfish behaviors can strongly make individuals more susceptible to infection (Gradito et al. 2024). This is something to consider carefully when exploring relationships between behaviors and parasite infection in natural settings. Our findings highlight the importance of studying wild animals harboring their natural parasite fauna and how the inclusion of a proxy such as body condition can help reveal hidden correlations between co-infection and behavior.

### Inhibitory avoidance learning

Contrary to our predictions, fish with more parasites were not negatively affected in the avoidance-learning task. From an ecological point of view, inhibitory avoidance learning is a relevant task, as these parasites would benefit from impairing the host’s ability to avoid predator attacks. We would expect parasites to impair cognitive abilities, making the host more susceptible to predation. One of the only other studies exploring the relationship between cognitive performance and parasites in fishes found that performance in a visual discrimination task was lower in Ambon damselfish (*Pomacentrus amboinensis)* infected both experimentally and naturally with ectoparasitic gnathiids (Crustacea: Isopoda), and also decreased with increasing endoparasite load (Binning et al. 2018). Since we did not perform an experimental infection, we cannot establish causality in our system. However, we could expect a trade-off between immune system efficiency and cognitive functions caused by long-term infection (Schleich et al. 2015; Barber et al. 2017). This trade-off has been experimentally tested with guppies (*Poecilia reticulata*), where selection for large brain size reduces innate immune responses (Kotrschal et al. 2016). In our study, we did not find any evidence of such a trade-off. Cognitive impairments due to parasitism may take longer to manifest and/or may only become evident during periods of intense cognitive demand. Fish were tested only twice over a short period of time, limiting our understanding of long-term impact of infection on cognition for this species. Further, since this infection is common, wild pumpkinseed sunfish may be more tolerant over time, and thus, detecting correlations between parasitism and cognition could be difficult. Factors other than body condition could be better suited to reveal hidden trade-offs in this case. For instance, adding immunity parameters could help reveal correlations between parasitism and cognition. More recently, a review argued that low performance does not necessarily indicate low cognitive abilities, which depend on individual, sex and environmental conditions (Bshary and Triki 2022). An alternative hypothesis could be that fish which are slower to learn may also be slower to identify which habitats pose a higher risk of infection: lower cognitive abilities may be the cause of infection rather than the effect (Barber et al. 2017). For instance, a recent study showed that some pumpkinseed individuals can detect and avoid infected conspecifics and habitats (Côté et al 2025). More studies with experimental infections are needed to better understand the relationship between cognition and infection.

### Other considerations

The use of seine nets as a collection method allowed us to randomly select behavioral phenotypes from the wild (Wilson et al. 1993). However, this included some very weak individuals: we experienced a relatively high mortality rate (26 fish) during the habituation period in the lab prior to testing. Thus, only relatively healthy fish were used in our experiments. This is a common phenomenon which presents a challenge to estimating the true costs of parasite infection in wild-caught individuals: those in the poorest health often do not survive in the lab long enough to be tested. Accordingly, only a few fish tested had extremely high parasite densities (one subject with 227 bass tapeworms). Clearly infections reach these levels in the wild. Thus, our results likely underestimate the true costs and behavioral changes induced by these parasite infections since severely affected individuals likely die prematurely in nature.

In nature, most hosts are infected with multiple parasite species at a time (Poulin 2007; Rigaud et al. 2010; Venter et al. 2022). For example, sunfish used in this study were all naturally parasitized with three or more species (*Proteocephalus ambloplites, Apophallus sp.,* and in much lower intensities, *Uvulifer sp*., *Clinostomum marginatum* and/or an unidentified nematode). This can lead to conflict between parasites with different life history strategies and/or developmental stages. Conflict between trophically transmitted parasites usually occurs when at least one of the two species is manipulative (Cezilly et al. 2014). For example, a study on copepods infected experimentally with a nematode (*Camallanus lacustris*) and a cestode (*Schistocephalus solidus*) showed that one parasite can reduce or sabotage host manipulation by another parasite from a different species (Hafer and Milinski 2016). Similarly, amphipods naturally infected with a manipulative trematode (*Microphallus papillorobustus*) do not show a strong behavioral alteration when they are also infected with a non-manipulative nematode (*Gammarinema gammari*), suggesting that the nematode potentially decreases manipulation by the trematode (Thomas et al. 2002). *Apophallus sp.* and *P. ambloplites* are both trophically transmitted parasites with different final hosts. *Apophallus sp.* reproduces in terrestrial animals that feed on infected fish. Conversely, *P. amblolites* reproduces in the gut of piscivorous fish like the small-mouth bass (*Micropterus dolomieu*) (McDaniel and Bailey 1974). We did not find any significant interactions between blackspot and internal parasite density for all traits. However, these two parasites benefit from increased host susceptibility to different predators, which may lead them to alter different aspects of host behavior. For instance, interactions between *P. ambloplites* and *Apophallus sp.* could alter the preferred position of the fish hosts in the water column. Even though blackspots caused by *Apophallus sp.* are more numerous on hosts, *P. ambloplites* are greater in terms of their biomass and have a clear physiological impact on sunfish, which could lead to manipulation through effects on physiology (Poulin 2013; Guitard et al. 2022; Sabbagh et al. 2024). Further research is crucial to disentangle the relationship between this co-infection and host behavior.

## Conclusion

Controlled infection experiments in the laboratory are critical for establishing the direction of causality in relationships among parasites, personality and cognitive traits. However, recreating natural conditions in a laboratory setting is often impossible, hampering ecological inferences. Thus, studies on wild populations may provide more realistic insights into the magnitude and consequences of individual variation in cognitive and personality traits across an infection gradient. We found that the relationship between parasite density and host personality depends on body condition in wild pumpkinseed sunfish. Body condition is an important measure of overall health that should be considered while studying wild populations as it has the potential to reveal otherwise hidden patterns. We suggest that the relationship between host personality and bass tapeworm density in sunfish could be explained by pathological response from the host, possibly making the fish more vulnerable for parasite transmission. Experimental parasite removal or infection are now needed to further assess the links between behavior and parasitism in this system. Experimental manipulation of parasite loads on free-living individuals could also be a useful approach to understand how wild animals experience co-infection (Payne et al. 2024). Our study shows the importance of including covariates to help reveal hidden correlations, especially in the context of parasitism.

## Data Availability

Analyses reported in this article can be reproduced using the data provided by Thelamon et al. (2025).
